# Biliverdin Reductase A Protects Lens Epithelial Cells against Oxidative Damage and Cellular Senescence in Age-Related Cataract

**DOI:** 10.1155/2022/5628946

**Published:** 2022-07-19

**Authors:** Yang Huang, Ying Liu, Siwei Yu, Wenzhe Li, Jinglan Li, Bo Zhao, Xin Hu, Haiying Jin

**Affiliations:** ^1^Department of Ophthalmology, Shanghai East Hospital, Tongji University School of Medicine, Shanghai, China; ^2^Department of Ophthalmology, Shanghai Electric Power Hospital, Shanghai, China; ^3^Department of Ophthalmology, Beijing Rehabilitation Hospital, Capital Medical University, Beijing, China; ^4^Department of Critical Care Medicine, Shanghai Electric Power Hospital, Shanghai, China; ^5^School of Ophthalmology & Optometry, Wenzhou Medical University, Zhejiang Province, China; ^6^Medical School of Chinese PLA, Beijing, China; ^7^Department of Ophthalmology, Huaihe Hospital, Henan University, Henan Province, China

## Abstract

Age-related cataract (ARC) is the common cause of blindness globally. Reactive oxygen species (ROS), one of the greatest contributors to aging process, leads to oxidative damage and senescence of lens epithelial cells (LECs), which are involved in the pathogenesis of ARC. Biliverdin reductase A (BVRA) has ROS-scavenging ability by converting biliverdin (BV) into bilirubin (BR). However, little is known about the protective effect of BVRA against ARC. In the present study, we measured the expression level of BVRA and BR generation in human samples. Then, the antioxidative property of BVRA was compared between the young and senescent LECs upon stress condition. In addition, we evaluated the effect of BVRA on attenuating H_2_O_2_-induced premature senescence in LECs. The results showed that the mRNA expression level of BVRA and BR concentration were decreased in both LECs and lens cortex of age-related nuclear cataract. Using the RNA interference technique, we found that BVRA defends LECs against oxidative stress via (i) restoring mitochondrial dysfunction in a BR-dependent manner, (ii) inducing heme oxygenase-1 (HO-1) expression directly, and (iii) promoting phosphorylation of ERK1/2 and nuclear delivery of nuclear factor erythroid 2-related factor 2 (Nrf2). Intriguingly, the antioxidative effect of BVRA was diminished along with the reduced BR concentration and repressed nuclear translocation of BVRA and Nrf2 in senescent LECs, which would be resulted from the decreased BVRA activity and impaired nucleocytoplasmic trafficking. Eventually, we confirmed that BVRA accelerates the G1 phase transition and prevents against H_2_O_2_-induced premature senescence in LECs. In summary, BVRA protects LECs against oxidative stress and cellular senescence in ARC by converting BV into BR, inducing HO-1 expression, and activating the ERK/Nrf2 pathway. This trial is registered with ChiCTR2000036059.

## 1. Introduction

Age-related cataract (ARC), the clouding crystalline lens, is a common cause of blindness globally [1]. ARC can be divided into three subtypes: age-related cortical cataract (ARC-C), age-related nuclear cataract (ARC-N), and age-related posterior subcapsular cataract (ARC-P) [2]. Although the pathogenesis of ARC remains unclear, aging is regarded as an independent risk factor [3]. Multiple studies, at present, have suggested a series of age-related changes in the lens including the depletion of antioxidants, the increase of lipid peroxidation, and alterations of posttranslational modification of soluble proteins, all of which give rise to aggregation of misfolded proteins and lens opacity [4, 5].

Lens epithelial cells (LECs), the most metabolically active component, are the first barrier of crystalline lens against hazardous external stimuli. The dysfunction of LECs is involved in the initiation and progression of ARC formation [6]. Cataractogenic stressors, e.g., hydrogen peroxide (H_2_O_2_), lead to excessive reactive oxygen species (ROS) accumulation and antioxidant depletion in LECs with age, which in turn promote oxidative stress and cellular apoptosis [7]. The contents of H_2_O_2_ in aqueous humor of ARC patients were approximately 20-folds higher than those of healthy subjects [8]. H_2_O_2_ exposure results in apoptosis by depleting glutathione (GSH) and decreasing superoxide dismutase (SOD) activity in LECs [9]. In addition, H_2_O_2_ treatment also induces similar morphological changes in *ex vivo* animal lenses compared with the lenses of ARC patients [10].

Cellular senescence, characterized as a permanent cell cycle arrest with increased senescence-associated *β*-galactosidase (SA-*β*-gal) activity and senescence-associated secretory phenotype (SASP) in response to cellular stresses, is a contributor to multiple age-related ocular disorders [11, 12]. Numerous studies have confirmed that oxidative stress is one of the most prominent risk factors in the development of aging phenotype [13, 14]. The exposure to a sublethal level of H_2_O_2_ results in the increased ROS level and premature senescence of LECs, which suggests a potential crosstalk between cellular senescence and oxidative stress in ARC formation [15]. Thus, restoration of the redox homeostasis may maintain LECs at a youthful stage and protects against lens opacity.

Biliverdin reductase A (BVRA) is an enzyme that rapidly converts biliverdin (BV) to bilirubin (BR). Recent studies confirmed that BVRA has ROS-scavenging ability by the production of BR, which is a robust endogenous antioxidant [16]. The antioxidative effect of BR depends profoundly on the redox cycle, in which BR is oxidized to BV by intracellular ROS, and BV is reduced back to BR by BVRA in turn [17]. Our previous study also confirmed that BR pretreatment prevents LECs from H_2_O_2_-induced oxidative insults [18]. In addition, BVRA promotes heme oxygenase-1 (HO-1) transcription through nuclear trafficking directly [19, 20] and activates nuclear factor erythroid 2-related factor 2 (Nrf2)/antioxidant response element (ARE) signaling via binding to ERK2 [21, 22]. The induction of HO-1 and activation of the ERK/Nrf2 pathway further fortify the capacity of BR against oxidative damage.

Despite the reported protective effect of BVRA on cellular oxidative stress by others, its antioxidative property in LECs and its role in regulating cellular senescence have not been studied. In the present study, we evaluated the BVRA expression levels and BR concentrations in human samples of ARC patients. Moreover, the protective role of BVRA against oxidative damage and cellular senescence was determined in LECs.

## 2. Materials and Methods

### 2.1. Study Participants

Forty-five LECs and lens cortex samples from ARC patients and fifteen control samples from healthy subjects were collected from Shanghai East Hospital. ARC patients were classified into three subgroups: ARC-C (*n* = 15 eyes), ARC-N (*n* = 15 eyes), and ARC-P (*n* = 15 eyes). The control samples were from patients who underwent clear lens extraction and multifocal intraocular lens implantation for presbyopia correction. The demographic data are listed in Table 1. The study was approved by the Institutional Review Board and Ethics Committee of Shanghai East Hospital. All procedures in the present study were in accordance with the tenets of Declaration of Helsinki, and written informed consent was provided from each donator.

All patients had a complete ophthalmic examination. Cataract grade for each patient was assessed using the Lens Opacity Classification System III (LOCS) with two individuals (H. Jin and Y. Huang). Subjects with a history of ocular trauma, intraocular surgery, glaucoma, uveitis, high myopia, retinitis pigmentosa, or systemic diseases, such as chronic inflammation and diabetic mellitus, were excluded.

### 2.2. LECs and Lens Cortex Preparation

The LECs were collected by anterior continuous curvilinear capsulorrhexis during phacoemulsification. Lens cortex was collected from the transparent cortex of controls and opaque cortex of ARC patients. All the procedures were performed by the same surgeon (H. Jin). Each sample was stored in a microtube and frozen at -80°C immediately for later extraction of mRNA detection.

### 2.3. Cell Culture

Male wild-type C57BL/6 mice (6-week-old, 20-25 g) were purchased from Beijing Long'An Animal Center. The animals were treated in accordance with the ARVO statement for the Use of Animals in Ophthalmic and Vision Research. The approach to obtain mouse LECs from native lens capsule tissue was consistent with our previous study [18]. Cells were cultured in Dulbecco's modified Eagle's medium containing 2% fetal bovine serum.

### 2.4. Two Types of LEC Aging Model *In Vitro*

For natural aging model, mouse LECs were passed weekly, and medium was changed twice a week. LECs at passages 4 and 15 were defined as young cells and senescent cells, respectively.

For H_2_O_2_-induced aging model, cells were incubated with 100 *μ*M H_2_O_2_ for 1 week. Fresh H_2_O_2_-containing medium was changed every day. Senescent cells were characterized as positive SA-*β*-gal staining and prolonged population doubling times.

### 2.5. Plasmids, RNA Interference, and Cell Transfection

The negative control small interfering RNA (siNC) and siRNA targeting BVRA (siBVRA) were synthesized by BoRui Co., Ltd (Beijing, China). The sequences of siRNA are listed in Table S1. The BVRA overexpression plasmid (pcDNA3.1-BVRA) and empty control plasmid (pcDNA3.1-NC) were established by cloning the cDNA of BVRA and negative control into pcDNA3.1 plasmid (V79020; Invitrogen, Shanghai, China), respectively. For transfection, cells were transfected with recombinant plasmids or siRNA by Lipofectamine 3000™ (L3000001; Invitrogen, Shanghai, China) according to the manufacturer's protocol.

### 2.6. BR Detection

The concentration of BR in whole cell lysates were measured by QuantiChrom™ Bilirubin Assay Kit (DIBR-180, BioAssay Systems, California, USA) according to the manufacturer's protocol. The color change was detected at 530 nm wavelength. The BR level was calculated as [OD_sample_ − OD_blank_]/[OD_calibration_ − OD_H2O_] × 5 mg/dL.

### 2.7. BVRA Assay

The BVRA activity assay was conducted by detecting the rate of BR converted from BV as previously described [23]. In brief, LEC cytosol (150 *μ*g of extracted protein) was incubated with 100 *μ*M BV (30891; Sigma-Aldrich, Shanghai, China) for 5 min and reacted in an NADPH-dependent manner (NADPH at a final concentration of 100 *μ*M) for 18 min at pH 8.7 at room temperature. The generation of BR was measured with a microplate reader (BioTec, Gene Company Ltd., USA) at 450 nm. BVRA activity was presented as units per milliliter with 1 unit of BVRA converting 1 nM BV to BR.

### 2.8. Cell Counting Kit-8

Cell counting kit-8 (CCK-8; Dojindo, Kumamoto, Japan) was used to determine the proliferation rate of LECs. After knocking down or overexpressing BVRA, cells were seeded in 96-well plates at a density of 5 × 10^3^ per well. 10 *μ*L of CCK-8 solution was added into each well at 48 h and 96 h, followed by incubating at 37°C for 3 h. The optical density was detected at 450 nm wavelength with a microplate reader.

### 2.9. Immunofluorescence Staining

5 × 10^4^/well LECs were seeded in 24-well plates and treated with 200 *μ*M H_2_O_2_ for 4 h. Then, samples were fixated and incubated with primary antibody against BVRA (sc-393385; Santa Cruz, 1 : 200) and goat anti-mouse IgG H&L (ab150113; Abcam, 1 : 5000). The reaction was detected using immunofluorescence microscope (Zeiss, German).

### 2.10. SA-*β*-Gal Staining

After being fixed with the fixing solution, cells were stained with SA-*β*-galactosidase staining kit (C0602, Beyotime, Shanghai, China) at 37°C overnight according to the manufacturer's protocol. SA-*β*-gal-positive cells were observed and counted by a microscope.

### 2.11. Flow Cytometry

The mitochondrial ROS level, mitochondrial membrane potential (MMP), apoptosis rate, and cell cycle were assessed by flow cytometry (BD FACSAria, San Jose, CA). For mitochondrial ROS detection, treated cells in various groups were incubated with 5 *μ*M MitoSOX Red probe (M36008, Thermo Fisher Scientific, China) at 37°C for 20 min. The fluorescence intensity was detected at 510 nm wavelength.

For MMP detection, treated cells were incubated with JC-1 solution (ab13850; Abcam, Shanghai, China) at 37°C for 30 min. The fluorescence intensity was detected at 488 nm and 575 nm wavelength.

Annexin V-FITC apoptosis staining kit (ab14085; Abcam, Shanghai, China) was used to measure the apoptotic rate of LECs. 1 × 10^5^ cells were incubated with 500 *μ*L of Annexin V binding buffer, followed by 5 *μ*L of Annexin V-FITC and 5 *μ*L of PI at room temperature in the dark for 5 min. The fluorescence intensity was detected at 488 nm wavelength.

Propidium iodide flow cytometry kit (ab139418; Abcam, Shanghai, China) was used for cell cycle analysis. 1 × 10^5^ cells were harvested, centrifuged, and resuspended in 200 *μ*L of propidium iodide and RNase staining solution at 37°C in the dark for 30 min. The fluorescence intensity was detected at 488 nm wavelength.

### 2.12. Coimmunoprecipitation (CoIP) Assay

Cell lysates were incubated with primary antibodies against BVRA (sc-393385; Santa Cruz Biotechnology, Inc., CA, USA), ERK2 (ab32081; Abcam, Shanghai, China) or normal mouse IgG (sc-2025; Santa Cruz Biotechnology, Inc., CA, USA) at 4°C overnight, followed by incubating with protein A/G Plus-Agarose beads (20423; Thermo Fisher Scientific, MA, USA) at 4°C for 2 h. The beads were then boiled for western blot analysis.

### 2.13. Quantitative Real-Time Polymerase Chain Reaction (qPCR)

The qPCR was performed as previously described [18]. Total RNA in each sample was extracted with TRIzol™ reagent, followed by RNA purification and RNA level calculation. Then, PCR reaction was performed by StepOne™ Real-Time PCR system with PowerTrack™ SYBR Green Master Mix (A46012; Thermo Fisher Scientific, Shanghai, China). The primer sequences are shown in Table S2. Relative gene expression levels were measured with the 2^(-*ΔΔ*Ct)^ method. GAPDH was used as a quantitative loading control.

### 2.14. Western Blot Analysis

Protein samples from LECs of each group were added to SDS-PAGE and blotted onto PVDF membrane. Primary antibodies against p21 (ab109199; Abcam, 1 : 1000), CDKN2A/p16^INK4*α*^ (ab211542; Abcam, 1 : 1000), Cyclin D1 (ab16663; Abcam, 1 : 1000), CDK4 (ab199728; Abcam, 1 : 1000), phosphorylated-ERK1/2 (ab201015; Abcam, 1 : 1000), ERK1/2 (ab184699; Abcam, 1 : 1000), BVRA (sc-393385; Santa Cruz, 1 : 1000), ERK2 (ab32081; Abcam, 1 : 1000), Nrf2 (ab92946; Abcam, 1 : 1000), HO-1 (ab68477; Abcam, 1 : 1000), GAPDH (ab8245; Abcam, 1 : 5000), and Histone H3 (ab1791; Abcam, 1 : 5000) were incubated with blots at 4°C overnight, followed by incubation with goat anti-mouse IgG H&L (ab150113; Abcam, 1 : 5000) or goat anti-rabbit IgG H&L (ab150077; Abcam, 1 : 5000) for 1 h at room temperature. GAPDH and Histone H3 were used as the quantitative loading control.

### 2.15. Statistical Analysis

The sample size of the recruited subject in the present study was calculated by PASS 15 software (NCSS, LLC. Kaysville, Utah, USA). Data were expressed as the mean ± standard error of the mean (SEM) and analyzed by GraphPad Prism 8 software (San Diego, CA, USA). Each experiment was performed for three individual times. The results were compared with the chi-square test or one-way ANOVA with the post hoc least significant difference test. *P* < 0.05 was regarded as statistical difference.

## 3. Results

### 3.1. Decreased Levels of BVRA and BR in the LECs and Lens Cortex of ARNC Patients

**Figure 1:**
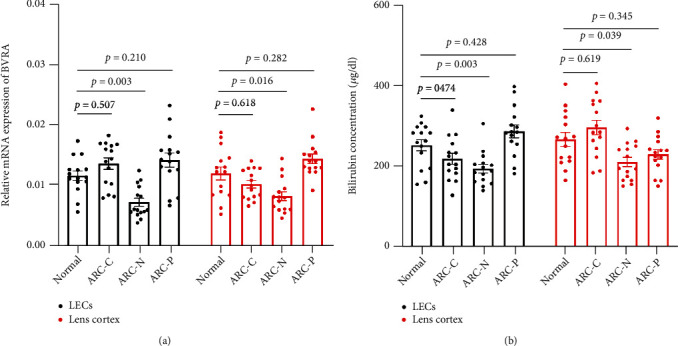
The relative mRNA expression of BVRA and BR concentration were decreased in LECs and lens cortex of ARC-N. The relative mRNA expression of BVRA (a) and BR concentration (b) in LECs and lens cortex among noncataract, ARC-C, ARC-N, and ARC-P were detected by qPCR and colorimetric method, respectively. *n* = 15 in each group. Data are shown as mean ± SEM, one-way ANOVA.

The relative mRNA expression levels of BVRA and BR concentration were both lower in LECs (*P* = 0.003, *P* = 0.030) and lens cortex (*P* = 0.016, *P* = 0.039) of ARC-N patients compared with the controls, respectively (Figures 1(a) and 1(b)). However, the data showed no difference in LECs and lens cortex among the controls, ARC-C, and ARC-P patients (all *P* > 0.05).

### 3.2. Aging-Dependent Differences in Redox Homeostasis by BVRA Silencing

**Figure 2:**
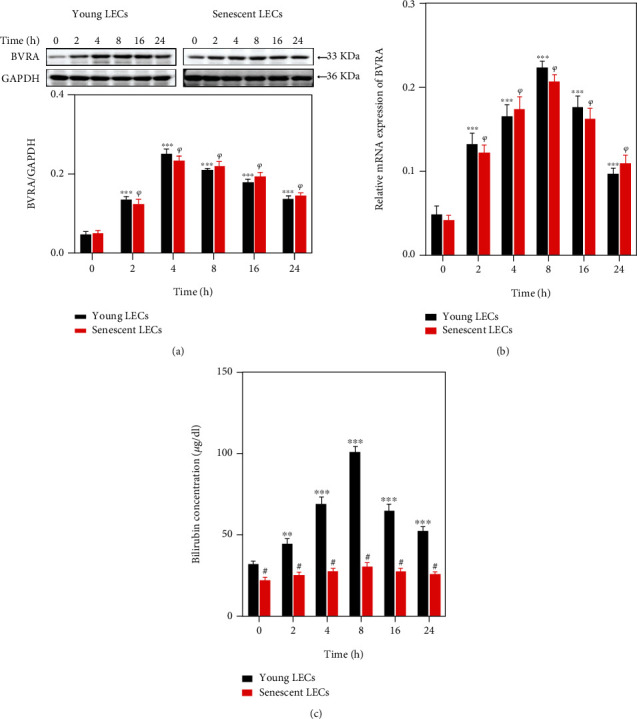
BVRA expression levels and BR production in young and senescent LECs under oxidative stress. The young and senescent LECs were exposed to 200 *μ*M H_2_O_2_ for various time points. (a) The representative bands of BVRA protein were shown by western blot analysis and semiquantified with GAPDH as reference. (b) The relative mRNA expressions of BVRA were measured by qPCR. (c) The intracellular BR concentrations were assessed by colorimetric method. Data are shown as mean ± SEM, one-way ANOVA, *n* = 3. ^∗∗^*P* < 0.01 and ^∗∗∗^*P* < 0.001, compared with young LECs without H_2_O_2_ damage. ^*ψ*^*P* < 0.05, compared with senescent LECs without H_2_O_2_ damage. Student's *t*-test, ^#^*P* < 0.05, compared with time-matched young LECs.

In order to elucidate the crosstalk among BVRA, oxidative stress, and cellular senescence, we first compared the expression pattern of BVRA in mRNA and protein levels and BR generation in both H_2_O_2_-damaged young and senescent LECs. At baseline, the BVRA expression levels were of no difference between the two types of cells, while the BR production was reduced in senescent LECs. In response to H_2_O_2_, the mRNA level of BVRA in both young and senescent cells reached the peak at 4 h in contrast to the baseline, while the protein level of BVRA and the intracellular BR concentration reached the peak at 8 h. BVRA mRNA and protein levels were of no difference between the two types of cells (Figures 2(a) and 2(b)). However, treatment with H_2_O_2_ dramatically upregulated BR generation in young LECs, but not in senescent cells (Figure 2(c)).

**Figure 3:**
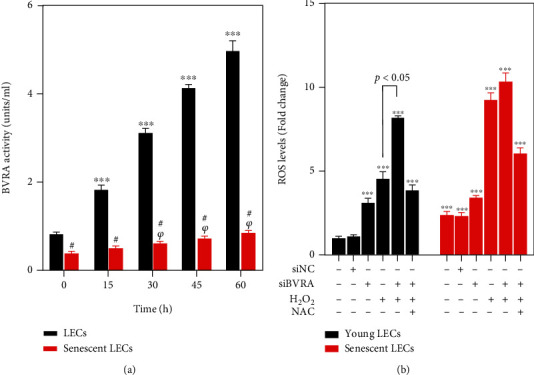
The effect of BVRA knockdown on intracellular ROS levels in young and senescent LECs upon stress conditions. (a) Young and senescent LECs were exposed to 200 *μ*M H_2_O_2_ for 15 min, 30 min, 45 min, and 60 min. Then, cell extract was prepared and BVRA activity was evaluated by detecting the rate of BR converted from BV in an NADPH-dependent manner at pH 8.5. (b) After being transfected with BVRA siRNA or NC siRNA, the young and senescent LECs were exposed to 200 *μ*M H_2_O_2_ for 24 h with or without 20 mM N-acetyl-L-cysteine (NAC). Intracellular ROS levels were detected by DCFH-DA staining. Data are shown as mean ± SEM, one-way ANOVA, *n* = 3. ^∗∗∗^*P* < 0.001, compared with young LECs without H_2_O_2_ damage. ^*ψ*^*P* < 0.05, compared with senescent LECs without H_2_O_2_ damage. Student's *t*-test, ^#^*P* < 0.05, compared with time-matched young LECs.

As BR is reduced from BV by BVRA, we further compared BVRA activity in both young and senescent LECs. At baseline, the BVRA activity was lower in senescent LECs. Treatment with H_2_O_2_ dramatically enhanced BVRA activity in young LECs, but not in senescent cells (Figure 3(a)).

For the role of BVRA in redox homeostasis, we evaluated the changes of intracellular ROS level by BVRA silencing. At baseline, the ROS level in senescent cells was about 3-fold higher than that in young cells. In young cells, the ROS level elevated more than 4-fold after knocking down BVRA, which is far more significant than that in senescent cells. Furthermore, BVRA silencing resulted in an enhanced sensitivity to H_2_O_2_ exposure in young LECs. On the contrary, there were no dramatic changes toward oxidative stress by the knockdown of BVRA in the senescent cells. In addition, pretreatment with 20 mM N-acetyl-L-cysteine (NAC), a robust ROS scavenger, for 2 h remarkably reduced the ROS levels in both young and senescent LECs in response to H_2_O_2_ after BVRA knockdown. All the results confirmed that BVRA is responsible for counteracting redox imbalance, especially in young LECs (Figure 3(b)).

### 3.3. Failure of BVRA Nuclear Trafficking Led to Hypoinduction of HO-1 in Senescent LECs under Oxidative Stress

**Figure 4:**
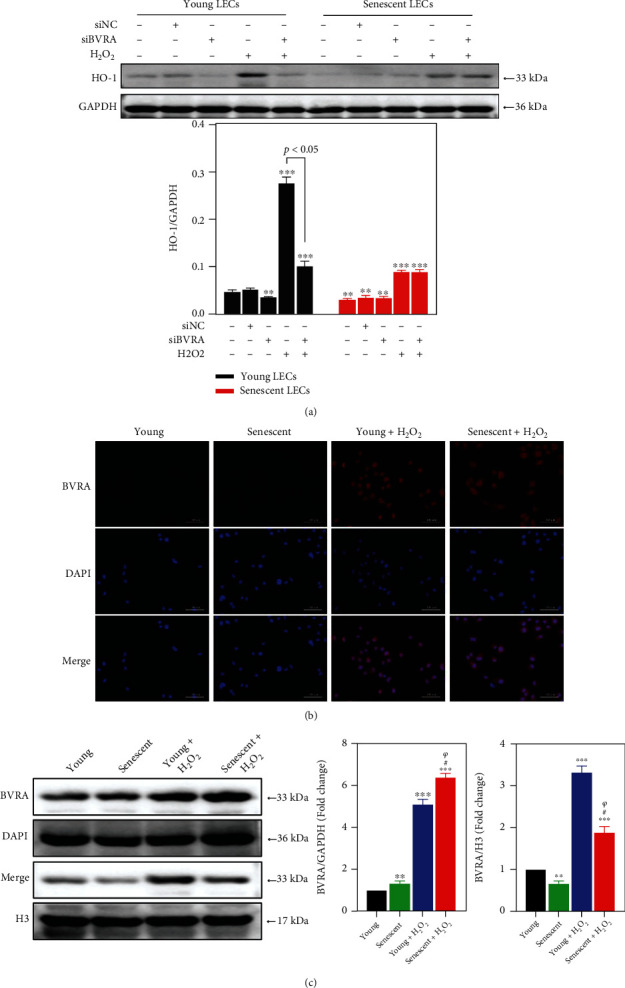
Failure of BVRA nuclear translocation led to hypoinduction of HO-1 in senescent LECs under oxidative stress. (a) After being transfected with BVRA siRNA or NC siRNA, the young and senescent LECs were exposed to 200 *μ*M H_2_O_2_ for 1 h. The relative protein expressions of HO-1 were analyzed by western blotting. (b) The young and senescent LECs were exposed to 200 *μ*M H_2_O_2_ for 4 h. Cells were immunostained with antibody against BVRA (red) and DAPI (blue). (c) The relative protein expressions of BVRA in the nucleus and cytoplasm were detected by western blot. Data are shown as mean ± SEM, *n* = 3, one-way ANOVA, ^∗∗^*P* < 0.01 and ^∗∗∗^*P* < 0.001, compared with young LECs. ^#^*P* < 0.05, compared with senescent LECs. ^*ψ*^*P* < 0.05, compared with H_2_O_2_-treated young LECs. Bar 100 *μ*m.

HO-1 shows cytoprotective and antioxidative effect in LECs [24]. HO-1 induction can be directly regulated by BVRA nuclear trafficking [20]. Thus, we detected the HO-1 expression in both young and senescent cells after silencing of BVRA to understand the aging-dependent differences in redox homeostasis. At baseline, the HO-1 level was lower in senescent LECs compared with that in young cells. H_2_O_2_ exposure led to a more obvious increase of HO-1 induction in young cells than in old cells. Meanwhile, HO-1 induction was notably suppressed by BVRA knockdown in young cells with or without H_2_O_2_ exposure. However, these effects were not observed in senescent LECs (Figure 4(a)).

Then, we evaluated the capacity for nuclear trafficking of BVRA under H_2_O_2_ exposure by immunofluorescence staining and western blot analysis. In young LECs, treatment with H_2_O_2_ resulted in a strong tendency for BVRA (red channel) to enter into the nucleus (blue channel). However, such phenomenon was abolished in senescent cells in response to H_2_O_2_ (Figure 4(b)). In contrast to the young LECs, BVRA protein levels in the nucleus were decreased in senescent cells at baseline. H_2_O_2_ exposure led to an upregulation of BVRA in the nucleus in young LECs, whereas BVRA was retained in the cytoplasm in senescent cells (Figure 4(c)). Thus, nuclear localization of BVRA was impaired in senescent LECs, which gave rise to the hypoinduction of HO-1 compared with the young cells upon stress conditions.

### 3.4. Failure of Nrf2 Nuclear Localization in Senescent LECs

**Figure 5:**
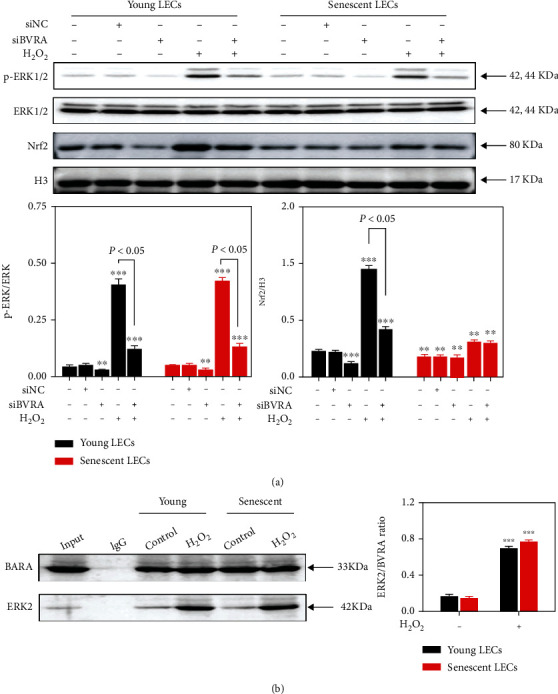
Failure of Nrf2 nuclear localization in senescent LECs. (a) The young and senescent LECs were transfected with BVRA siRNA or NC siRNA, followed by 200 *μ*M H_2_O_2_ exposure for 1 h. The effect of BVRA knockdown on the relative expression of phosphorylated-ERK1/2 and Nrf2 was assessed by western blot. (b) The young and senescent LECs were exposed to 200 *μ*M H_2_O_2_ for 4 h. The binding capacity of BVRA and ERK2 in LECs was determined by CoIP assay. Data are shown as mean ± SEM, *n* = 3, one-way ANOVA, ^∗∗^*P* < 0.01 and ^∗∗∗^*P* < 0.001, compared with young LECs.

It has been reported that BVRA-ERK2 binding induces ERK1/2 phosphorylation [21]. The phosphorylated-ERK1/2 (p-ERK1/2) activates Nrf2 nuclear localization, followed by transcription of ARE genes, including HO-1 [22, 25]. To further explore the antioxidative effect of BVRA on young and senescent LECs, we detected the expression levels of p-ERK1/2 and Nrf2 after BVRA knockdown by western blot. The data revealed no aging-dependent differences on p-ERK1/2 in LECs. H_2_O_2_ exposure led to an increase of ERK1/2 phosphorylation. Silencing of BVRA downregulated the expression of p-ERK1/2 in both young and senescent LECs upon H_2_O_2_ damage. Interestingly, the Nrf2 level in the nucleus at baseline was lower in senescent LECs compared with that in young cells. H_2_O_2_ exposure resulted in a dramatic nuclear translocation of Nrf2 in young cells than in old cells. Meanwhile, Nrf2 nuclear delivery was notably suppressed by BVRA knockdown in young cells exposed to H_2_O_2_. However, these effects were not observed in senescent cells. (Figure 5(a)).

Then, we assessed the interaction between BVRA and ERK in response to oxidative stress by CoIP assay. As shown in Figure 5(b), H_2_O_2_ exposure for 4 h resulted in an enhanced interaction between BVRA and ERK2 compared with the control group in both young and senescent LECs. All the above-mentioned results indicated that BVRA promotes ERK/Nrf2 activation in the presence of oxidants, while the decreased nuclear Nrf2 levels in senescent LECs may have resulted from the repressed capacity of nuclear delivery.

### 3.5. The Impaired Nucleocytoplasmic Trafficking toward H_2_O_2_ Damage in Senescent LECs

**Figure 6:**
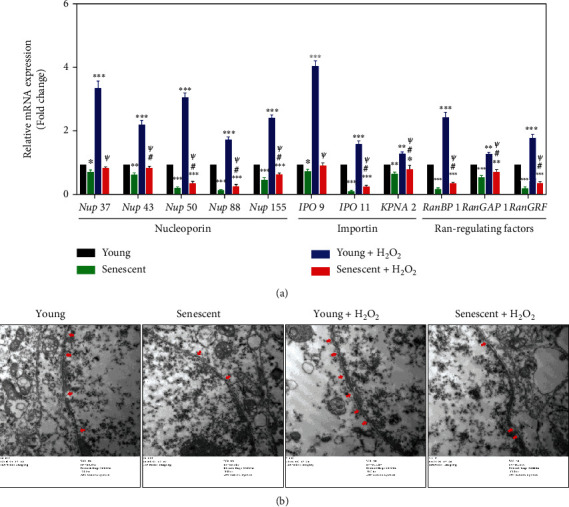
The impaired nucleocytoplasmic trafficking in senescent LECs under oxidative stress. The young and senescent LECs were exposed to 200 *μ*M H_2_O_2_ for 4 h. (a) The nucleocytoplasmic trafficking gene expressions were measured with qPCR. (b) NPCs (red arrows) in young and senescent LECs under oxidative stress were observed by a transmission electron microscope. Data are shown as mean ± SEM, *n* = 3, one-way ANOVA, ^∗^*P* < 0.05, ^∗∗^*P* < 0.01, and ^∗∗∗^*P* < 0.001, compared with young LECs. ^#^*P* < 0.05, compared with senescent LECs. ^*ψ*^*P* < 0.05, compared with H_2_O_2_-treated young LECs.

To clarify the mechanism of the impaired capacity for nuclear translocation of BVRA and Nrf2 in senescent LECs under oxidative stress, we analyzed the relative mRNA expression levels of several selected nucleocytoplasmic trafficking genes in young and senescent cells by qPCR. As shown in Figure 6(a), five selected nucleoporin genes (*Nup 37*, *Nup 43*, *Nup 50*, *Nup 88*, and *Nup 155*), three selected importin genes (*IPO 9*, *IPO 11*, and *KPNA2*), and three selected Ran-regulating factor genes (*RanBP1*, *RanGAP1*, and *RanGRF*) were all decreased in senescent LECs compared to young LECs. Moreover, H_2_O_2_ exposure gave rise to a more significant expression of nucleocytoplasmic trafficking genes in young cells, but not in senescent cells.

As nuclear pore complexes (NPCs) consist of nucleoporins, we further observed NPCs in young and senescent LECs toward H_2_O_2_ treatment by a transmission electron microscope. The data showed that the number of NPCs decreased in senescent LECs compared to young LECs. Moreover, H_2_O_2_ exposure increased the number of NPCs in young cells, but not in senescent cells (Figure 6(b)). All the data indicated that the nucleocytoplasmic trafficking is impaired in senescent LECs, by which the nuclear delivery of BVRA and Nrf2 would be prohibited.

### 3.6. BVRA Prohibited Mitochondrial Dysfunction in a BR-Dependent Manner

**Figure 7:**
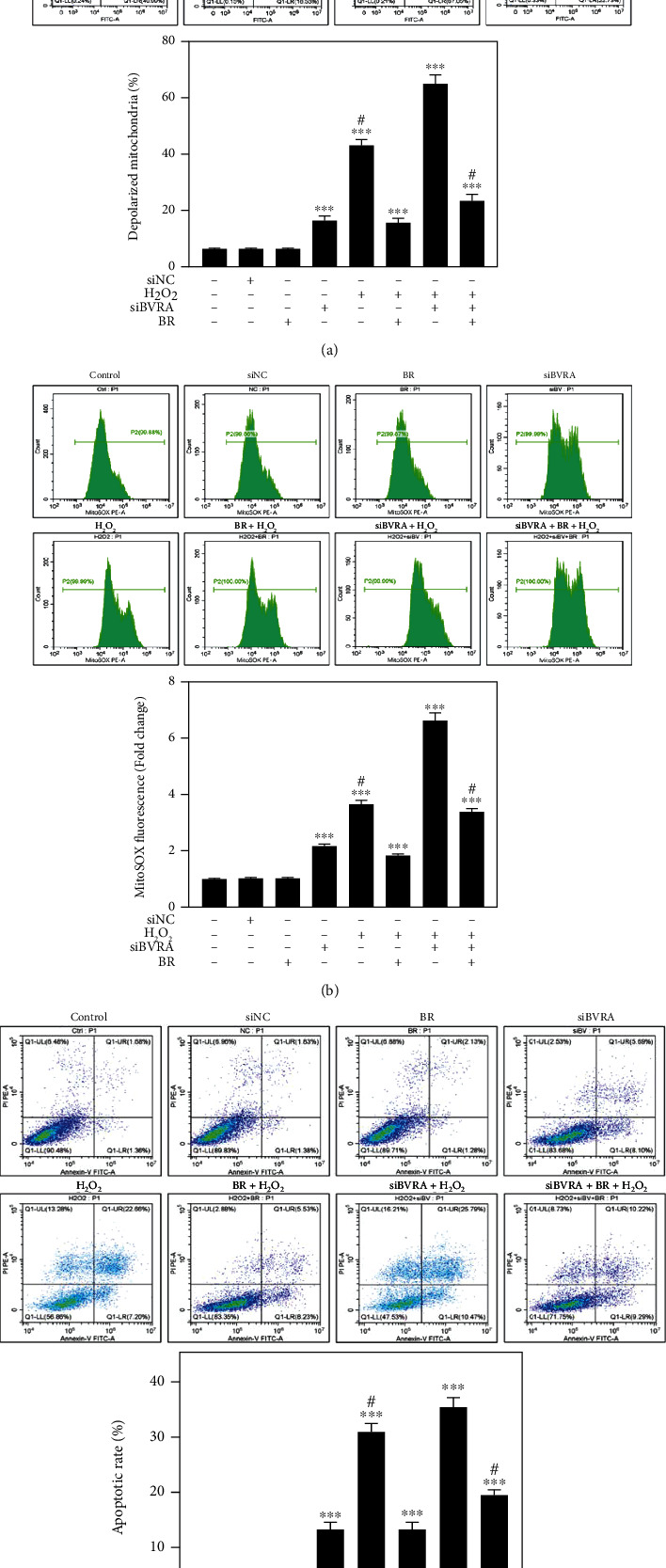
Mitochondrial dysfunction induced by BVRA depletion under oxidative stress was rescued by BR. LECs were transfected with BVRA siRNA. Then, cells were pretreated with 20 *μ*M BR for 2 h before exposure to 200 *μ*M H_2_O_2_ for 24 h. (a) The representative diagram of mitochondrial membrane potential determined by JC-1 staining. (b) Mitochondrial ROS levels were detected by MitoSOX probe. (c) The apoptotic rates of LECs were assessed by Annexin V-FITC assay. Data are shown as mean ± SEM. One-way ANOVA, ^∗∗^*P* < 0.01 and ^∗∗∗^*P* < 0.001, compared with the control group. ^#^*P* < 0.05 compared with the BVRA siRNA-transfected cells exposed to H_2_O_2_.

As mitochondrial dysfunction results in redox imbalance under oxidative stress, we further evaluated the effect of BR on mitochondrial membrane potential, mitochondrial ROS level, and apoptotic rate in BVRA silenced LECs. JC-1 was used to probe the changes of mitochondrial membrane potential. MitoSOX probe and Annexin V-FITC were used for measuring mitochondrial ROS levels and apoptotic rates, respectively. As a result, knockdown of BVRA induced an increase of mitochondrial membrane depolarization, mitochondrial ROS accumulation, and apoptosis in H_2_O_2_-damaged LECs, which were all restored by BR pretreatment partially (Figures 7(a)–7(c)). All these data demonstrated that BVRA protects LECs against mitochondrial dysfunction upon stress conditions in a BR-dependent manner.

### 3.7. BVRA Prevented Premature Senescence against Oxidative Stress in LECs

**Figure 8:**
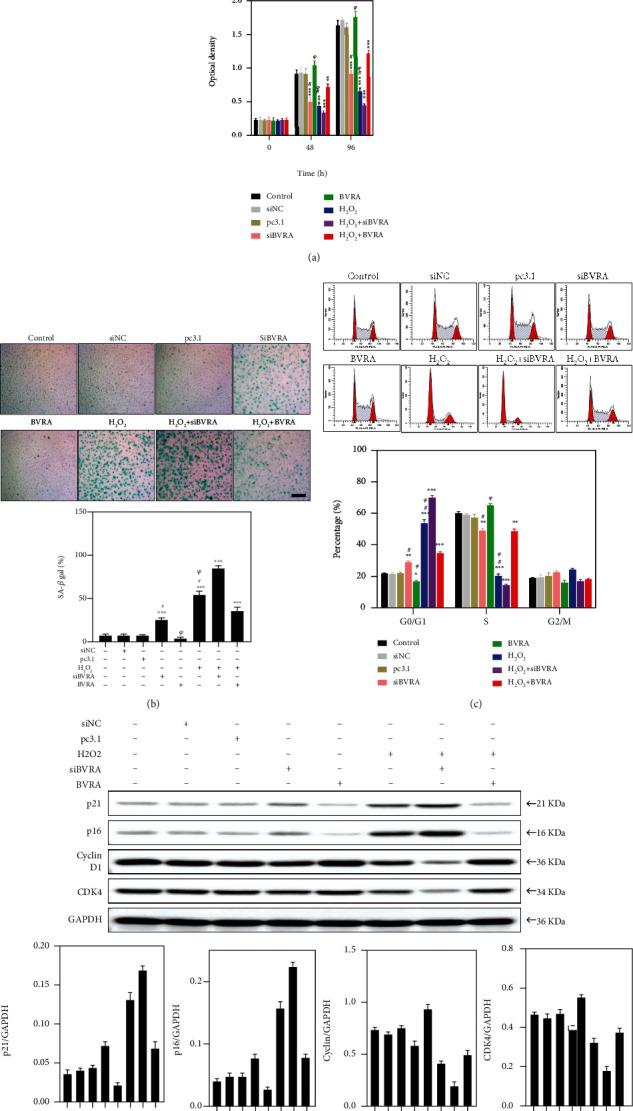
BVRA protects LECs against oxidative stress-induced cellular senescence. (a) After overexpressing or knocking down BVRA, LECs were incubated with H_2_O_2_ for 96 h. The morphological changes of young LECs were observed at 48 h and 96 h by a microscope. The cell proliferation was determined by CCK-8 assay at 48 h and 96 h. (b) BVRA overexpressed or silenced LECs were incubated with 100 *μ*M H_2_O_2_ for 7 days. The percentage of senescent cells was analyzed by SA-*β*-gal staining at day 7. (c) The effects of BVRA on the relative expression of cell cycle regulators (p21, p16^INK4*α*^, cyclin D1, and CDK4) at day 4 were determined by western blot. (d) The effects of BVRA on cell cycle progression in different groups at day 4. Data are shown as mean ± SEM. One-way ANOVA, ^∗∗^*P* < 0.01 and ^∗∗∗^*P* < 0.001, compared with the control group. ^#^*P* < 0.05 compared with the BVRA siRNA-transfected cells exposed to H_2_O_2_. ^*ψ*^*P* < 0.05 compared with the pcDNA3.1-BVRA-transfected cells exposed to H_2_O_2_. Bar 200 *μ*m.

Eventually, we evaluated the effect of BVRA on prevention of cellular senescence. As shown in Figure 8(a), siBVRA transfection and H_2_O_2_ exposure both repressed cell proliferation at 48 h and 96 h. In H_2_O_2_-exposed LECs, knockdown of BVRA further induced a decrease of cell number, while BVRA overexpression dramatically restores the cell number. By SA-*β*-gal staining, it was obvious that BVRA knockdown and H_2_O_2_ exposure both promote the expression of SA-*β*-gal, a senescence marker, in LECs at day 7. In H_2_O_2_-exposed LECs, knockdown of BVRA further increased the percentage of SA-*β*-gal-positive cells, while BVRA overexpression maintains LECs at a youthful stage (Figure 8(b)).

We further detected the expression levels of p21 and p16^INK4*α*^, which are dramatically upregulated in senescent cells and implicate cell cycle arrest [26]. In contrast to the control group, silencing of BVRA increases the p21 and p16^INK4*α*^ levels. In H_2_O_2_-exposed LECs, BVRA knockdown further resulted in higher levels of p21 and p16^INK4*α*^, while BVRA overexpression decreased p21 and p16^INK4*α*^ levels (Figure 8(c)). To figure out the role of BVRA on cell cycle progression, we performed flow cytometry. In H_2_O_2_-exposed LECs, knockdown of BVRA further increased the number of cells in the G_0_/G_1_ phase and decreased the number of cells in the S phase, while BVRA overexpression restored the cell number to the basic level (Figure 8(d)). Furthermore, in H_2_O_2_-exposed LECs, BVRA depletion led to the downregulation of cyclin D1 and CDK4 levels, which confirmed the G_0_/G_1_ cell cycle arrest [27]. Nevertheless, BVRA overexpression restored cyclin D1 and CDK4 levels (Figure 8(c)). All these data strongly suggested that BVRA accelerates the G1 phase transition and prevents against H_2_O_2_-induced premature senescence in LECs.

## 4. Discussion

**Figure 9:**
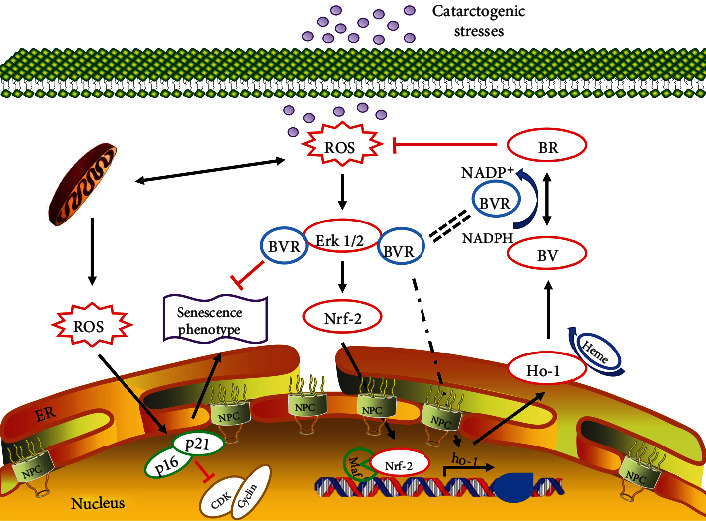
The effect of BVRA on preventing cellular senescence against oxidative stress in LECs. Cellular senescence, an important risk factor for ARC formation, is closely associated with redox imbalance in LECs. Cataractogenic stresses give rise to intracellular ROS accumulation, antioxidant depletion, and cell aging in LECs. To prevent cellular senescence against oxidative stress, the BVRA-mediated antioxidative defense system takes effect in three manners. First, BVRA protects LECs from mitochondrial dysfunction and eliminates ROS in a BR-dependent manner by converting BV into BR. Second, enhanced nuclear trafficking of BVRA directly promotes HO-1 expression upon oxidation. Third, BVRA activates ERK/Nrf2 signaling by promoting phosphorylation of ERK1/2 and Nrf2 nuclear localization. However, the antioxidative effect of BVRA was diminished in senescent LECs, which would be resulted from the decreased enzymatic activity of BVRA and the repressed nucleocytoplasmic trafficking.

In this study, we confirmed that the relative mRNA expression level of BVRA and BR concentration are decreased in both LECs and lens cortex of ARNC patients compared with the normal subjects. Moreover, BVRA prevents cellular senescence against oxidative stress in three manners: (i) restoring mitochondrial dysfunction by converting BV into BR, (ii) inducing HO-1 expression directly, and (iii) increasing phosphorylation of ERK1/2, which further activates the Nrf2/ARE pathway. However, the cytoprotective property of BVRA was diminished in senescent LECs, which would be resulting from the decreased BVRA activity and the impaired nucleocytoplasmic trafficking. To sum up, the present study indicated that the BVRA-mediated antioxidative defense system protects lens transparency against oxidative stress and cellular senescence (Figure 9).

Chronic oxidative stress and aging are the independent risk factors for ARC [28]. Crystalline lens has a powerful antioxidant defense system to counteract intracellular ROS and maintain redox homeostasis [29]. Nevertheless, ROS overwhelm the cellular antioxidant capacity in the lens with advancing age, giving rise to ARC eventually. According to clinical manifestations, ARC can be divided into three subtypes, among which oxidative stress is closely related to ARC-N [30]. ARC-N is characterized by ROS accumulation and GSH depletion in the lens nucleus [31]. In the present study, we found that the relative mRNA level of BVRA and BR concentration in both LECs and lens cortex of ARC-N were decreased compared with those of noncataract subjects. The lipophilic BR protects the cell membrane against lipid peroxidation [32], while the hydrophilic GSH primarily protects water-soluble proteins against oxidation and aggregation [33]. Thus, our data indicated that the BVRA/BR axis may be a novel antioxidative defense system in the lens tissue and would have complementary cytoprotective effect together with GSH on maintaining lens transparency.

The primary physiological function of BVRA is the generation of lipophilic BR. The antioxidative effect of the BVRA/BR axis is significantly amplified by BVRA in the BV-BR cycle. BR is oxidized to BV in response to free radicals, and BV is rapidly reduced back to BR by BVRA in turn [17, 34]. According to our previous results, BV/BR redox pair can prohibit H_2_O_2_-induced apoptosis via suppressing intracellular ROS levels and restoring GSH and SOD contents in LECs [18]. In the present study, we further confirmed that BR pretreatment rescues mitochondrial dysfunction induced by BVRA depletion under oxidative stress, which suggests that BVRA protects LECs in a BR-dependent manner.

Apart from the reductase function, BVRA protects against oxidants by inducing HO-1 expression directly [35, 36]. The domain of BVRA has a leucine zipper structure, which can recognize and bind to two activator protein (AP-1) sites in HO-1 promoter region [19]. Under multiple stimuli, cytosolic BVRA is translocated into the nucleus and promotes HO-1 transcription [37]. HO-1 is the rate-limiting enzyme for heme degradation, which produces three by-products, biliverdin, carbon monoxide, (CO) and free iron (Fe^2+^) [38]. HO-1 has anti-inflammatory, antioxidative, and antiapoptotic effects in various diseases [39, 40]. Previously, we verified that loss of HO-1 activity gives rise to endoplasmic reticulum stress and ROS accumulation in the lens, followed by early onset of nuclear cataract in mice [24]. In addition, our series of studies also indicated that the HO-1/CO axis protects LECs against oxidative stress *in vitro* [9, 41, 42]. In the present work, our data suggest that BVRA acts as the ROS scavenger by enhancing its nuclear localization under H_2_O_2_ exposure, which promotes HO-1 expression in young LECs.

Nrf2, a redox-sensitive transcription factor, acts as the chief regulator of the body's antioxidant defense system [43]. Under basal conditions, Nrf2 binds to Kelch-like ECH-associated protein 1 (Keap-1) and retains in the cytoplasm [44]. In response to oxidants, Nrf2 is released from Keap-1 and translocated into the nucleus. As a result, Nrf2 combined with ARE and promotes the transcription of various detoxification and antioxidant enzymes (e.g., HO-1, NADPH: quinone oxidoreductase 1 (NQO-1), glutamate-cysteine ligase-modifier subunits (GCLM), and SOD) [45, 46]. Previously, we verified the cytoprotective role of Nrf2 via inhibiting endoplasmic reticulum stress in LECs [47]. Intriguingly, Nrf2 activation can also be regulated by BVRA. Baranano et al. observed the downregulation of Nrf2 gene expression in BVRA silenced cells [48]. BVRA can bind to ERK2 and promotes phosphorylation of ERK1/2 [21], which is a significant activator of the Keap-1/Nrf2/ARE pathway [49]. Several studies suggest that ERK phosphorylation facilitates the dissociation of Nrf2 from Keap-1, allowing nuclear delivery of Nrf2 and gene expression of ARE [50, 51]. Our data also confirmed that knockdown of BVRA leads to a decreased level of ERK1/2 phosphorylation and prohibits the nuclear delivery of Nrf2. Thus, BVRA activates the ERK/Nrf2 pathway via promoting phosphorylation of ERK1/2.

In contrast to young LECs, BVRA cannot protect against stresses in senescent cells in this work. At baseline, the ROS level in senescent LECs was higher than that in young cells. In the presence of oxidants, BVRA knockdown dramatically increased intracellular ROS level in young LECs, but not in senescent cells. In addition, silencing of BVRA attenuated HO-1 induction and Nrf2 nuclear localization in H_2_O_2_-exposed young LECs, while these effects were not observed in senescent cells. Apart from decreased BVRA enzymatic activity, these aging-dependent differences in the oxidative response may be elucidated by the impaired functional nucleocytoplasmic trafficking in old cells. Senescent cells were characterized by hyporesponsiveness to multiple stimuli, which may be due to the aging-related nuclear barrier [52–54]. The nucleocytoplasmic transport process is highly organized and is performed by a variety of transport machineries that include NPCs, karyopherins (importins and exportins), and Ran proteins [55]. NPC is a protein complex composed of nucleoporins, which allows large molecules to shuttle between the nucleus and cytoplasm [56]. Ran proteins control the karyopherins/cargo protein assembly and disassembly [57]. In the present study, the expression of nucleocytoplasmic trafficking genes and the number of NPCs were all decreased in senescent LECs compared to young LECs. Moreover, H_2_O_2_ exposure resulted in an increase of nucleocytoplasmic trafficking gene levels and the number of NPCs in young cells, but not in senescent cells. All the data indicated that the nucleocytoplasmic trafficking is impaired in senescent LECs upon stress conditions, by which the nuclear delivery of BVRA and Nrf2 would be prohibited.

Cellular senescence is presented as cell cycle arrest, increased expression of SA-*β*-gal, mitochondrial dysfunction, proinflammatory response, and telomere shortening [58]. Excessive intraocular ROS level is a predominant factor inducing cellular senescence, which is demonstrated by the fact that antioxidants delay or prevent cell aging [59]. In the present work, we confirmed that BVRA can effectively attenuate premature senescence in H_2_O_2_-treated LECs by SA-*β*-gal staining. Furthermore, the data also revealed that BVRA restores the increased levels of p21 and p16^INK4*α*^, the increased number of cells in G_0_/G_1_ phase, and the downregulated levels of cyclin D1 and CDK4 under stress conditions. All the results implicated that BVRA accelerates the G1 phase transition and prevents premature senescence against oxidative stress in LECs.

## 5. Conclusions

In conclusion, this study reveals that BVRA has antioxidative and antisenescent properties on crystalline lens and H_2_O_2_-treated LECs. It is suggested that the underlying mechanism is through restoring mitochondrial dysfunction by converting BV into BR, inducing HO-1 expression directly, and activating the ERK/Nrf2/ARE pathway. These findings indicate that pharmacological agents targeting BVRA may serve as a potential protective strategy for ARC patients.

## Figures and Tables

**Table 1:** Demographic characteristics of patients with or without ARC.

	Noncataract (*n* = 15)	ARC-C (*n* = 15)	ARC-N (*n* = 15)	ARC-P (*n* = 15)	*P* value
Age (years)	57.20 ± 3.90	58.27 ± 3.56	60.13 ± 4.75	59.80 ± 4.51	0.202^a^
Gender (male/female)	7/8	8/7	6/9	7/8	0.911^b^

^a^One-way ANOVA; ^b^chi-square test. ARC-C: age-related cortical cataract; ARC-N: age-related nuclear cataract; ARC-P: age-related posterior subcapsular cataract.

## Data Availability

All data generated and analyzed in the present study are available from the corresponding author on reasonable request.

## Supplementary Materials

Supplementary Materials Table S1: the sequences of siRNA are listed. Table S2: the primer pairs for qPCR are listed.

## References

[1] Assi L., Chamseddine F., Ibrahim P. (2021). A global assessment of eye health and quality of life: a systematic review of systematic reviews. *JAMA Ophthalmology*.

[2] Liu Y. C., Wilkins M., Kim T., Malyugin B., Mehta J. S. (2017). Cataracts. *Lancet*.

[3] Faranda A. P., Shihan M. H., Wang Y., Duncan M. K. (2021). The aging mouse lens transcriptome. *Experimental Eye Research*.

[4] Periyasamy P., Shinohara T. (2017). Age-related cataracts: role of unfolded protein response, Ca^2+^ mobilization, epigenetic DNA modifications, and loss of Nrf2/Keap1 dependent cytoprotection. *Progress in Retinal and Eye Research*.

[5] Wei Z., Hao C., Huangfu J., Srinivasagan R., Zhang X., Fan X. (2021). Aging lens epithelium is susceptible to ferroptosis. *Free Radical Biology & Medicine*.

[6] Xu J., Li D., Lu Y., Zheng T. Y. (2021). A*β* monomers protect lens epithelial cells against oxidative stress by upregulating CDC25B. *Free Radical Biology & Medicine*.

[7] Hu S., Su D., Sun L. (2020). High-expression of ROCK1 modulates the apoptosis of lens epithelial cells in age-related cataracts by targeting p53 gene. *Molecular Medicine*.

[8] Spector A., Garner W. H. (1981). Hydrogen peroxide and human cataract. *Experimental Eye Research*.

[9] Huang Y., Ma T., Ye Z. (2018). Carbon monoxide (CO) inhibits hydrogen peroxide (H_2_O_2_)-induced oxidative stress and the activation of NF-*κ*B signaling in lens epithelial cells. *Experimental Eye Research*.

[10] Hernebring M., Adelöf J., Wiseman J., Petersen A., Zetterberg M. (2021). H_2_O_2_-induced cataract as a model of age-related cataract: lessons learned from overexpressing the proteasome activator PA28*αβ* in mouse eye lens. *Experimental Eye Research*.

[11] Roy A. L., Sierra F., Howcroft K. (2020). A blueprint for characterizing senescence. *Cell*.

[12] Sreekumar P. G., Hinton D. R., Kannan R. (2020). The emerging role of senescence in ocular disease. *Oxidative Medicine and Cellular Longevity*.

[13] Finkel T., Holbrook N. J. (2000). Oxidants, oxidative stress and the biology of ageing. *Nature*.

[14] Sun K., Yang P., Zhao R., Bai Y., Guo Z. (2018). Matrine attenuates D-galactose-induced aging-related behavior in mice via inhibition of cellular senescence and oxidative stress. *Oxidative Medicine and Cellular Longevity*.

[15] Chen M., Zhang C., Zhou N., Wang X., Su D., Qi Y. (2021). Metformin alleviates oxidative stress-induced senescence of human lens epithelial cells via AMPK activation and autophagic flux restoration. *Journal of Cellular and Molecular Medicine*.

[16] Mancuso C. (2021). Biliverdin reductase as a target in drug research and development: facts and hypotheses. *Free Radical Biology & Medicine*.

[17] Ryter S. W. (2022). Heme oxygenase-1: an anti-inflammatory effector in cardiovascular, lung, and related metabolic disorders. *Antioxidants (Basel)*.

[18] Huang Y., Li J., Li W., Ai N., Jin H. (2022). Biliverdin/bilirubin redox pair protects lens epithelial cells against oxidative stress in age-related cataract by regulating NF-kappaB/iNOS and Nrf2/HO-1 pathways. *Oxidative Medicine and Cellular Longevity*.

[19] Ahmad Z., Salim M., Maines M. D. (2002). Human biliverdin reductase is a leucine zipper-like DNA-binding protein and functions in transcriptional activation of heme oxygenase-1 by oxidative stress. *The Journal of Biological Chemistry*.

[20] Tudor C., Lerner-Marmarosh N., Engelborghs Y., Gibbs P. E. M., Maines M. D. (2008). Biliverdin reductase is a transporter of haem into the nucleus and is essential for regulation of HO-1 gene expression by haematin. *The Biochemical Journal*.

[21] Lerner-Marmarosh N., Miralem T., Gibbs P. E., Maines M. D. (2008). Human biliverdin reductase is an ERK activator; hBVR is an ERK nuclear transporter and is required for MAPK signaling. *Proceedings of the National Academy of Sciences of the United States of America*.

[22] Shi Y., Sun Y., Sun X. (2018). Up-regulation of HO-1 by Nrf2 activation protects against palmitic acid- induced ROS increase in human neuroblastoma BE(2)-M17 cells. *Nutrition Research*.

[23] Kim S. Y., Kang H. T., Choi H. R., Park S. C. (2011). Biliverdin reductase A in the prevention of cellular senescence against oxidative stress. *Experimental & Molecular Medicine*.

[24] Huang Y., Ye Z., Yin Y. (2021). Cataract formation in transgenic HO-1 G143H mutant mice: involvement of oxidative stress and endoplasmic reticulum stress. *Biochemical and Biophysical Research Communications*.

[25] Wong S. Y., Tan M. G., Wong P. T., Herr D. R., Lai M. K. P. (2016). Andrographolide induces Nrf2 and heme oxygenase 1 in astrocytes by activating p38 MAPK and ERK. *Journal of Neuroinflammation*.

[26] Beausejour C. M., Krtolica A., Galimi F. (2003). Reversal of human cellular senescence: roles of the p53 and p16 pathways. *The EMBO Journal*.

[27] Wu C. L., Shan T. D., Han Y. (2021). Long intergenic noncoding RNA 00665 promotes proliferation and inhibits apoptosis in colorectal cancer by regulating miR-126-5p. *Aging (Albany NY)*.

[28] Wang S., Yu M., Yan H., Liu J., Guo C. (2022). MiR-34a-5p negatively regulates oxidative stress on lens epithelial cells by silencing GPX3 - a novel target. *Current Eye Research*.

[29] Yeum K. J., Taylor A., Tang G., Russell R. M. (1995). Measurement of carotenoids, retinoids, and tocopherols in human lenses. *Investigative Ophthalmology & Visual Science*.

[30] Truscott R. J. (2005). Age-related nuclear cataract--oxidation is the key. *Experimental Eye Research*.

[31] Lim J. C., Grey A. C., Zahraei A., Donaldson P. J. (2020). Age-dependent changes in glutathione metabolism pathways in the lens: new insights into therapeutic strategies to prevent cataract formation-a review. *Clinical & Experimental Ophthalmology*.

[32] Sedlak T. W., Saleh M., Higginson D. S., Paul B. D., Juluri K. R., Snyder S. H. (2009). Bilirubin and glutathione have complementary antioxidant and cytoprotective roles. *Proceedings of the National Academy of Sciences of the United States of America*.

[33] Giblin F. J. (2000). Glutathione: a vital lens antioxidant. *Journal of Ocular Pharmacology and Therapeutics*.

[34] Vasavda C., Kothari R., Malla A. P. (2019). Bilirubin links heme metabolism to neuroprotection by scavenging superoxide. *Cell Chemical Biology*.

[35] Wang J., de Montellano P. R. (2003). The binding sites on human heme oxygenase-1 for cytochrome P450 reductase and biliverdin reductase. *The Journal of Biological Chemistry*.

[36] Kravets A., Hu Z., Miralem T., Torno M. D., Maines M. D. (2004). Biliverdin reductase, a novel regulator for induction of activating transcription factor-2 and heme oxygenase-1. *The Journal of Biological Chemistry*.

[37] Kapitulnik J., Maines M. D. (2009). Pleiotropic functions of biliverdin reductase: cellular signaling and generation of cytoprotective and cytotoxic bilirubin. *Trends in Pharmacological Sciences*.

[38] Rochette L., Zeller M., Cottin Y., Vergely C. (2018). Redox functions of heme oxygenase-1 and biliverdin reductase in diabetes. *Trends in Endocrinology and Metabolism*.

[39] Althunibat O. Y., Abduh M. S., Abukhalil M. H., Aladaileh S. H., Hanieh H., Mahmoud A. M. (2022). Umbelliferone prevents isoproterenol-induced myocardial injury by upregulating Nrf2/HO-1 signaling, and attenuating oxidative stress, inflammation, and cell death in rats. *Biomedicine & Pharmacotherapy*.

[40] Park J. S., Saeed K., Jo M. H. (2022). LDHB deficiency promotes mitochondrial dysfunction mediated oxidative stress and neurodegeneration in adult mouse brain. *Antioxidants*.

[41] Ma T., Chen T., Li P. (2016). Heme oxygenase-1 (HO-1) protects human lens epithelial cells (SRA01/04) against hydrogen peroxide (H_2_O_2_)-induced oxidative stress and apoptosis. *Experimental Eye Research*.

[42] Huang Y., Ye Z., Ma T. (2018). Carbon monoxide (CO) modulates hydrogen peroxide (H_2_O_2_)-mediated cellular dysfunction by targeting mitochondria in rabbit lens epithelial cells. *Experimental Eye Research*.

[43] Osama A., Zhang J., Yao J., Yao X., Fang J. (2020). Nrf2: a dark horse in Alzheimer's disease treatment. *Ageing Research Reviews*.

[44] Lao Y., Wang Y., Chen J. (2022). Synthesis and biological evaluation of 1,2,4-triazole derivatives as potential Nrf2 activators for the treatment of cerebral ischemic injury. *European Journal of Medicinal Chemistry*.

[45] Zhou H. S., Hu L. B., Zhang H. (2020). Design, synthesis, and structure-activity relationships of indoline-based Kelch-like ECH-associated protein 1-nuclear factor (erythroid-derived 2)-like 2 (Keap1-Nrf2) protein-protein interaction inhibitors. *Journal of Medicinal Chemistry*.

[46] Wu A. G., Yong Y. Y., Pan Y. R. (2022). Targeting Nrf2-mediated oxidative stress response in traumatic brain injury: therapeutic perspectives of phytochemicals. *Oxidative Medicine and Cellular Longevity*.

[47] Ma T. J., Lan D. H., He S. Z. (2018). Nrf2 protects human lens epithelial cells against H2O2-induced oxidative and ER stress: the ATF4 may be involved. *Experimental Eye Research*.

[48] Baranano D. E., Rao M., Ferris C. D., Snyder S. H. (2002). Biliverdin reductase: a major physiologic cytoprotectant. *Proceedings of the National Academy of Sciences of the United States of America*.

[49] Deng S., Liu S., Jin P. (2021). Albumin reduces oxidative stress and neuronal apoptosis via the ERK/Nrf2/HO-1 pathway after intracerebral hemorrhage in rats. *Oxidative Medicine and Cellular Longevity*.

[50] Zipper L. M., Mulcahy R. T. (2003). Erk activation is required for Nrf2 nuclear localization during pyrrolidine dithiocarbamate induction of glutamate cysteine ligase modulatory gene expression in HepG2 cells. *Toxicological Sciences*.

[51] Park J. Y., Kang K. A., Kim K. C., Cha J. W., Kim E. H., Hyun J. W. (2013). Morin induces heme oxygenase-1 via ERK-Nrf2 signaling pathway. *Journal of Cancer Prevention*.

[52] Ryu S. J., An H. J., Oh Y. S., Choi H. R., Ha M. K., Park S. C. (2008). On the role of major vault protein in the resistance of senescent human diploid fibroblasts to apoptosis. *Cell Death and Differentiation*.

[53] Kim S. Y., Ryu S. J., Ahn H. J., Choi H. R., Kang H. T., Park S. C. (2010). Senescence-related functional nuclear barrier by down-regulation of nucleo- cytoplasmic trafficking gene expression. *Biochemical and Biophysical Research Communications*.

[54] Lim I. K., Won Hong K., Kwak I. H., Yoon G., Park S. C. (2000). Cytoplasmic retention of p-Erk1/2 and nuclear accumulation of actin proteins during cellular senescence in human diploid fibroblasts. *Mechanisms of Ageing and Development*.

[55] Cautain B., Hill R., de Pedro N., Link W. (2015). Components and regulation of nuclear transport processes. *The FEBS Journal*.

[56] Stoffler D., Fahrenkrog B., Aebi U. (1999). The nuclear pore complex: from molecular architecture to functional dynamics. *Current Opinion in Cell Biology*.

[57] Izaurralde E., Kutay U., von Kobbe C., Mattaj I. W., Görlich D. (1997). The asymmetric distribution of the constituents of the Ran system is essential for transport into and out of the nucleus. *The EMBO Journal*.

[58] Childs B. G., Durik M., Baker D. J., van Deursen J. M. (2015). Cellular senescence in aging and age-related disease: from mechanisms to therapy. *Nature Medicine*.

[59] Munoz-Espin D., Serrano M. (2014). Cellular senescence: from physiology to pathology. *Nature Reviews Molecular Cell Biology*.

