# Supplementary material for: Biliverdin Reductase A Protects Lens Epithelial Cells against Oxidative Damage and Cellular Senescence in Age-Related Cataract
Source: Oxid Med Cell Longev. 2022 Jul 19;2022:5628946. doi: 10.1155/2022/5628946 (PMC9325611; doi:10.1155/2022/5628946)
Supplement: Supplementary Materials — Table S1: the sequences of siRNA are listed. Table S2: the primer pairs for qPCR are listed. [file 5628946.f1.docx]

**Supplementary Materials**

**Table S1:** **The sequences of RNA interference**

|  | Sense (5’→3’) | Antisense (5’→3’) |
| --- | --- | --- |
| siBVRA | CUUACAGUGUUGACAUCUAAA | UAGAUGUCAACACUGUAAGGG |
| NC siRNA | UUCUCCGAACGUGUCACGUTT | ACGUGACACGUUCGGAGAATT |

**Table S2: The sequences of primer pairs for qPCR**

| Gene | Forward (5’→3’) | Reverse (5’→3’) |
| --- | --- | --- |
| BVRA (human) | TAATGCTGGCAAGCACGT | GGTCTTTCCCCACCACTTCT |
| GAPDH (human) | GGAGCGAGATCCCTCCAAAAT | GGCTGTTGTCATACTTCTCATGG |
| BVRA (mouse) | GAAAGGGAGAGTCCTGCATGA | CTGGCTGTGAAGCGAAGAGAT |
| Nup 37 (mouse) | TACACCGTGGATTGCGAAGAT | CCTGAAACGTACACATGCCAA |
| Nup 50 (mouse) | GGAGTTGACTGACAGGAACTG | CCTCCGCTATCAGATTCAAATCC |
| Nup 88 (mouse) | CCTAACCACGTCGTGTTCTTG | CGAAGACCAGGTTTCTCGTCG |
| Nup 155 (mouse) | AAACGCGGGGAGGCTTATC | CTGACAGCAAACCTGGTCCT |
| IPO 9 (mouse) | GCACAGGGATTGAAGGAGGC | TGCCAGGTGAACACCAAATTC |
| IPO 11 (mouse) | TGTGCCAGTCCTTTTATGAGC | CCTCTCGCCTTCAATAACACCT |
| KPNA2 (mouse) | ATGTCCACGAACGAGAATGCT | AAGGAGCTGACGTTTCTTCTTTT |
| RanBP1 (mouse) | CGAGGACCATGATACTTCCACA | CCTCCAGCGTTTTAATTTCTTGC |
| RanGAP1 (mouse) | CTCGAAGCTCTACGATTGGAGG | TGGTGGGATCTCAGACCGAAG |
| RanGRF (mouse) | CAGAAACTGTCCACTGTTCGG | GTCACAGGATGGCAGAAAACT |
| GAPDH (mouse) | TGGATTTGGACGCATTGGTC | TTTGCACTGGTACGTGTTGAT |
